# ADULT CHILDREN‘S SINGLEHOOD AND AGING PARENTS’ PSYCHOLOGICAL WELL-BEING IN SOUTH KOREA

**DOI:** 10.1093/geroni/igae098.2478

**Published:** 2024-12-31

**Authors:** Mihye Lim, Hyo Jung Lee

**Affiliations:** Yonsei University of Human Ecology, Goyang-si, Kyonggi-do, Republic of Korea; Yonsei University, Seoul, Republic of Korea

## Abstract

Guided by a life course perspective, this study aims to examine the linkages between the marital status of adult children as an indicator of the transition to adulthood and their parents’ psychological well-being, considering how parental socioeconomic resources play a role in this association. We conducted a series of regression models using data from the 2018 Korean Longitudinal Study of Ageing (KLOSA, n=3112 aging parents who had at least one child over 35 years old). Consistent with the exposure model, having an unmarried son was associated with poorer parental psychological well-being, irrespective of parental income and education. However, parental education partially explained the association between daughter’s marital status and parental psychological well-being, suggesting that parental well-being regarding adult children’s marital status may depend on child’s gender and parental education level. This study has implications for the intergenerational relationships in collectivist and patrilineal Eastern cultural context. Aging parents’ psychological well-being ties to their adult children’s marital status based on the social norm regarding the transition to adulthood, and particularly sons’ marital status than that of daughters’. Also, aging parents may have different views on daughter’s marital status depending on their educational attainment. In sum, based on the concept of ‘linked-lives’ and stress process model, this study provides an extended understanding of intergenerational relations in later life in Korea. Clinicians and family professionals should consider well-being in old age associated with adult children’s life transitions and gender in the cultural context.

